# Hyoscyamus

**Published:** 1888-08

**Authors:** Hamilton Evans


### HYOSCYAMUS.

This drug is indicated in those cases in which the nervous system is especially affected. The disorders calling for its use are not so deep-seated as those for which Belladonna would be suitable, nor do we notice the cerebral hyperæmia characteristic of the latter. On the other hand, even in its own sphere, the nervous system, it is inferior in intensity to Stramonium, while resembling it in some respects. Its symptoms resemble in some points those of delirium tremens, for which it is sometimes indicated. Among mental symptoms, we notice loss of consciousness, sometimes complete, sometimes partial. The patient may answer a question properly, but immediately becomes unconscious again. Sexual excitement sometimes prevails, with its natural concomitants. Dilatation of the pupils, with dim or perverted vision, is a frequent symptom; and visual hallucinations are not uncommon, the patient seeing, with great distinctness, though only with his mind's eye, objects or persons which are not present.

With regard to the convulsive attack, we may notice that the muscles are affected in two ways—sometimes being a subject to sudden jerks of short duration, and sometimes to long-lasting spasms. The muscles may be affected singly or in sets, or the whole body may be rigid, as in tetanus.

Sudden falling to the ground with shrieks and convulsions, as in epilepsy, would indicate this remedy, of which we may further add that it appears to be especially suitable for children, old men, and the victims of intoxicating beverages.

HAMILTON EVANS.

